# 3-day intervention program for family members of hikikomori sufferers: A pilot randomized controlled trial

**DOI:** 10.3389/fpsyt.2022.1029653

**Published:** 2023-01-09

**Authors:** Hiroaki Kubo, Hiromi Urata, Motohiro Sakai, Shunsuke Nonaka, Junji Kishimoto, Kazuhiko Saito, Masaru Tateno, Keiji Kobara, Daisuke Fujisawa, Naoki Hashimoto, Yuriko Suzuki, Yoko Honda, Tomohiro Nakao, Kotaro Otsuka, Shigenobu Kanba, Toshihide Kuroki, Takahiro A. Kato

**Affiliations:** ^1^Department of Neuropsychiatry, Graduate School of Medical Sciences, Kyushu University, Fukuoka, Japan; ^2^Department of Psychiatry, Faculty of Medicine, University of Miyazaki, Miyazaki, Japan; ^3^Faculty of Education, University of Miyazaki, Miyazaki, Japan; ^4^School of Child Psychology, Tokyo Future University, Tokyo, Japan; ^5^Center for Clinical and Translational Research, Kyushu University, Fukuoka, Japan; ^6^Aiiku Research Institute, Imperial Gift Foundation Boshi-Aiiku-Kai, Tokyo, Japan; ^7^Department of Neuropsychiatry, Sapporo Medical University, Sapporo, Japan; ^8^Shimane Prefectural Counseling Center for Physical and Mental Health, Shimane, Japan; ^9^Department of Neuropsychiatry, Keio University School of Medicine, Tokyo, Japan; ^10^Department of Psychiatry, Hokkaido University Graduate School of Medicine, Sapporo, Japan; ^11^Department of Mental Health Policy, National Institute of Mental Health, National Center of Neurology and Psychiatry, Tokyo, Japan; ^12^Fukuoka City Mental Health and Welfare Center, Fukuoka, Japan; ^13^Department of Neuropsychiatry, School of Medicine, Iwate Medical University, Iwate, Japan; ^14^Department of Clinical Psychology Practice, Graduate School of Human-Environment Studies, Kyushu University, Fukuoka, Japan

**Keywords:** pathological social withdrawal (hikikomori), mental health first aid (MHFA), community reinforcement and family training (CRAFT), family intervention, RCT (randomized controlled trial)

## Abstract

**Backgrounds:**

Hikikomori, pathological social withdrawal, is becoming a crucial mental health issue in Japan and worldwide. We have developed a 3-day family intervention program for hikikomori sufferers based on Mental Health First Aid (MHFA) and Community Reinforcement and Family Training (CRAFT). This study aims to confirm the effectiveness of the 3-day program by a randomized controlled trial.

**Methods:**

This study was registered on the UMIN Clinical Trials Registry (UMIN000037289). Fifteen parents were assigned to the treat as usual (TAU) group (TAU only; Age Mean, 65.6; *SD*, 7.8), and 14 to the Program group (program + TAU; Age Mean, 67.9; *SD*, 8.6). This study was discontinued due to the COVID-19 pandemic; the recruitment rate was 36.3% of our target sample size of 80.

**Results:**

Perceived skills improved temporally and stigma temporally worsened in the TAU group. Confidence decreased and attitude showed no change in both groups. Aggressive behaviors of hikikomori sufferers were significantly worsened in the Program group; however, no serious domestic violence was reported. In the TAU group, Avoidance and irregular life patterns were improved. Activity levels were worsened in both groups. Two participants (16.7%) in the Program group and one participant (7.7%) in the TAU group reported actual behavioral changes (e.g., utilizing support).

**Conclusion:**

We could not draw general conclusions on the effectiveness of the program due to the study discontinuation. Nevertheless, this study indicates the necessity for revision of the program to improve family members’ confidence in engaging with hikikomori sufferers, with safer approaching by families.

## Introduction

Pathological social withdrawal (hereafter, hikikomori) is a condition avoiding social participation and staying at home almost every day for 6 months or longer (1). Hikikomori was originally observed in Japan, but is now reported worldwide (2–5). Hikikomori sufferers rarely seek help by themselves; the role of family members is critical for intervention. However, family members have difficulties in approaching hikikomori sufferers and are in great distress (3). Thus, we have developed an intervention program based on Mental Health First Aid (MHFA) and Community Reinforcement and Family Training (CRAFT) for family members to acquire skills in approaching hikikomori sufferers (6, 7). MHFA provides knowledge and skills about mental health problems based on the 5-step principles to encourage professional supports (8, 9). CRAFT includes positive communication skills and functional analysis based on cognitive behavioral therapy to improve relationship between hikikomori sufferers and their family members (10). Combination of these is expected to allow family members to approach their hikikomori sufferers more safely and effectively, and we have already reported preliminary effectiveness of the program with single-arm trials (6, 7). The purpose of this study is to confirm the effectiveness of the 3-day program by a randomized controlled trial (RCT).

## Method

### Study design

This study was registered on the UMIN Clinical Trials Registry (UMIN-CTR) (UMIN000037289^1^). The study was a single-center, open-label, randomized trial, and allocation results were open to both participants and program providers. Participants were family members living with hikikomori sufferers. Participants were assigned in a 1:1 ratio to the standard intervention (family support) plus 3-day program (Program group) or the standard intervention only (TAU group) according to the randomization procedure described in Randomization.

### Participants

All the participants written informed consent for the participation of the study. The eligibility criteria were as follows; (1) living with hikikomori sufferers (2) participant receiving standard hikikomori family support (treat as usual; TAU) (3) age 20 years or older (4) hikikomori duration of their hikikomori sufferer was at least 6 months. The exclusion criteria were as follows; (1) participants have learned MHFA program previously, (2) hikikomori sufferers had continuously used the support institutions within the past 6 months, (3) difficulty in reading or writing Japanese, (4) difficulty in attending the program continuously due to a serious physical or psychiatric symptoms, (5) daily violence from the hikikomori sufferers, (6) the intervention is likely to cause danger to the participants or their family due to severe aggression of hikikomori sufferers, (7) the intervention is likely to cause hikikomori sufferers’ suicidal ideation or self-harming behavior.

Standard intervention (TAU) in this study is defined as continuous consultation or advice provided to a family member in person or by telephone at a hikikomori support institution such as mental health welfare center, hikikomori community support center, family association, youth support station, non-profit organization, community health center, medical institution, consultation center, social welfare council, or school.

Candidates were recruited through hikikomori support institutions including Kyushu University Hospital and related medical institutions, Fukuoka City Mental Health and Welfare Center, Fukuoka Hikikomori Community Support Center, hikikomori family associations, and public health centers in Fukuoka city. Candidates who applied to the study were contacted by phone to confirm eligibility and exclusion criteria. Recruitment began in June 2019; however, the study was discontinued in December 2020 due to COVID-19 pandemic in Japan.

### Randomization

Assignment was performed by the co-author (JK), a biostatistician in the Center for Clinical and Translational Research, Kyushu University. Randomization was assured because of his complete independence of program providers. Participants were assigned to the Program group or the TAU group immediately after the baseline interview to ascertain eligibility criteria and informed consent. Assignment included a 2 × 2 stratification with family avoidance by hikikomori sufferers and hikikomori duration (≥ 24 months/< 24 months) as allocation factors. The study was an open-label randomized trial, and allocation was open to both participants and program providers.

### Interventions

For the Program group, a program consisted of three sessions (3-day program) was implemented after informed consent. During the program, participants continued the standard intervention. The program was group family classes conducted fortnightly and each session was 180 min. Programs were conducted over a five-course period from June 2019 through March 2021, with group sizes ranging from 3 to 8 participants. All programs were conducted by a MHFA trainer psychiatrist TAK and two MHFA instructors, clinical psychologists (HK and HU). No follow-up sessions were held after the program; evaluations were conducted by mailed questionnaires.

Participants in the TAU group continued the standard intervention and were only evaluated *via* mailed questionnaires at the same time as the Program group. As an ethical view, participants in the TAU group were allowed to attend the program after completion of all evaluations if they wished.

No evaluation or intervention was directly conducted for their hikikomori sufferers (i.e., child) in either group.

### Program contents

The 3-day program was designed to provide family members of behavioral skills using scenario role-playing as well as lectures so that they can make appropriate approaches in response to hikikomori sufferers.

The program was a partially revised version of which we developed based on MHFA and CRAFT (7). The number of sessions, the intervals, and the duration per session were identical; content was added and the order of the content was rearranged. We added two contents; active listening was added to the first session which was conducted in the 5-day program (6). Active listening is an essential part of MHFA for establishing safer relationship between parents and their child (8, 9). We also added small-step approaches which is fundamental method of CRAFT (7, 10). Regarding the content-order rearrangement, the functional analysis that was conducted in the first session of the previous program was moved to the second session. No other program content was changed; however, the overall program content was increased due to adding the program content. The 3-day program content is shown in Table 1.

**TABLE 1:** Contents of 3-day program.

**Session 1**
Knowledge on mental health and therapeutic approach for hikikomori and the MHFA five-step approach was introduced. After the lecture, a pair of participants alternately performed a role-play of active listening based on MHFA principle (i.e., Step 2 of MHFA; listen non-judgmentally). Homework: Goal setting, considering and conducting daily communication such as greetings, invitation for playful events, call for help of domestic chores, and so on.
**Session 2**
Functional analysis based on CRAFT were introduced, including a workshop. After the lecture, a pair of participants alternately performed a role-play scenario of a parent and a hikikomori sufferer based on MHFA. Homework: Thinking about the MHFA-based approach to encourage hikikomori sufferers to utilize professional support. If possible, participants were required to practice the approach actually.
**Session 3**
Workshop on positive and small-stepped communication (e.g., non-judgmental, non-hostile, empathic, warmth approaches) based on MHFA and CRAFT was introduced. Review and discussion (Q and A) of the entire program were also conducted.

### Outcomes

Self-administered questionnaires were implemented at four time points: within 1 month of the program (T1; baseline), immediately after the program (T2), 2 months after the program (T3), and 6 months after the program (T4) for the Program group. For TAU group, questionnaires were administered at the same time as the Program group. All the questionnaires were mailed to the participants, and participants were asked to return them. We have collected the information on the participants (parents) themselves and their evaluation of hikikomori sufferers (child), as shown below.

### Primary

#### Perceived skill

We measured the perceived skills of approaching to hikikomori sufferers based on the five-steps of the MHFA, as evaluated in our previous studies (6, 7). We presented a vignette case of a hikikomori sufferer (Mr. A) and asked how well respondent would offer help based on the MHFA as a parent of the case using five-point scale indicating from 0 (absolutely no) to 4 (absolutely yes). In the previous study, the question consisted of nine items; in this study, a new MHFA item on active listening (“listening to Mr. A without criticizing him”) was added. Skills associated with CRAFT including small-step approaches were not evaluated in this case vignette questionnaire. Score ranges from 0 to 40, with higher scores indicating more MHFA related skills perceived by respondents. Reliability and validity of this questionnaire have not been confirmed; several researches reporting effects on MHFA utilized this questionnaire (11, 12).

#### Secondary outcomes

We measured the confidence associated with MHFA, stigma toward mental health problems, depressive symptoms and stress responses among participants, and problematic and adaptive behaviors in hikikomori sufferers. These measures were identical to our previous studies (6, 7).

#### Confidence

For confidence, the original 6-item scale was administered (6, 7). Based on the MHFA, six questions on a 5-point scale from 0 (not confident at all) to 4 (very confident). Score ranges from 0 to 24, with higher scores indicating more confident in dealing with people expressing depressive symptoms. Reliability and validity of this questionnaire have not been confirmed; several researches reporting effects on MHFA utilized this questionnaire (11, 12).

#### Stigma toward mental health problems

For stigma, the Japanese version of the 12-item Link’s Devaluation-Discrimination Scale was administered (13). Respondents are required to evaluate stigma level on community, which is thought to reflect the stigma of respondents. This scale is consisted of 12 questions with a 4-point scale from 1 (strongly disagree) to 4 (strongly agree). Score ranges from 12 to 48; the higher the score, the higher the stigma the respondent feels. Reliability and validity of the Japanese version scale were confirmed.

#### Depressive symptoms of participants

Participants’ depressive symptoms were assessed using the Japanese version of the Patient Health Questionnaire (PHQ-9) (14, 15). This scale evaluates the severity of depressive symptoms within the last 2 weeks using nine questions with a 4-point scale from 0 (not at all) to 3 (nearly every day). Score ranges from 0 to 27; the scores of 5, 10, 15, and 20 represents mild, moderate, moderately severe, and severe depression, respectively. Reliability and validity of the Japanese version scale were confirmed.

#### Stress response of participants

The Stress Response Scale-18 (SRS-18) was used to assess participants’ psychological stress responses (16). This scale is consisted of 18 items and has three factors as sub-scales: (1) Depression–Anxiety, (2) Irritability–Anger, and (3) Helplessness (six items, respectively). Respondents are required to answer their stress responses within 3 days with a 4-point scale from 0 (not at all) to 3 (strongly agree). Total score ranges from 0 to 54; More than 9 among males and 11 among females of total score indicate mediate stress response. Reliability and validity of the scale were confirmed.

#### Problematic behaviors of hikikomori sufferers

The Hikikomori Behavior Checklist (HBCL) was used to assess the problematic behaviors of hikikomori sufferers (17). This scale is consisted of 45 items and has 10 factors as sub-scales: (1) Aggressive behavior (eight items), (2) Social anxiety (four items), (3) Obsessive–compulsive behavior (four items), (4) Avoidance from family members (five items), (5) Depression (four items), (6) Absence of activities of daily living (six items), (7) Incomprehensible maladapted behavior (five items), (8) Absence of social participation (three items), (9) Decreased activity (three items), and (10) Irregular life pattern (three items). Respondents were asked regarding behavior within the last 3 months with a 4-point scale from 0 (not at all) to 3 (strongly agree). Total score ranges from 0 to 135, with higher score indicating problematic behavior among hikikomori sufferers. Reliability and validity of the scale were confirmed.

#### Adaptive behaviors of hikikomori sufferers

The Adaptive Behaviors Scale for Hikikomori (ABS-H) was used for hikikomori sufferers’ adaptive behaviors (18). This scale is consisted of 26 items and has four factors as sub-scales: (1) Interaction (14 items), (2) Family (four items), (3) Value (four items), and (4) Social participation (four items). Respondents were asked to answer regarding the behaviors of their hikikomori sufferers within the last 3 months using a 4-point scale from 0 (almost never) to 3 (almost always). Total score ranges from 0 to 78, with higher score indicating adaptive behavior among hikikomori sufferers. Reliability and validity of the scale were confirmed.

#### Attitude toward hikikomori sufferers

In addition, we conducted the Family Attitude Scale (FAS) to measure participants’ negative attitudes toward hikikomori sufferers (19). The FAS is a scale to measure expressed emotion (EE) and consists of 30 items. Respondents are asked to indicate how often each statement occurs on a 5-point scale from 0 (never) to 4 (every day). Score ranges from 0 to 120, with higher scores indicating more negative attitudes such as criticism and burden. The Japanese version of the FAS has been validated (19).

Besides these self-administered questionnaires, participants were asked to report actual behavioral changes such as social participation and adverse events including self-harm and aggressive behaviors among hikikomori sufferers. In the present study, two couples participated in each group; we incorporated maternal report because mothers tend to spend more time with hikikomori sufferers in many hikikomori cases.

#### Sample size estimation

Based on the results of previous pilot studies (6, 7), the sample size was calculated. Regarding changes (primary outcome) in the MHFA-related perceived skills scores at 2 months after the program; we assumed the Program group scored 3.5 and the TAU group 1.0, with a standard deviation of 4.5, α = 0.05 (two-sided), Power = 0.80, and the ratio of the participants was 1:1, the sample size required to obtain significance was estimated to be 70. Assuming dropout occurrence, 80 participants are considered to be reasonable.

### Statistical analysis

In this study, statistical analysis was performed with reference to previous RCTs reporting the efficacy of MHFA (20, 21). Data on self-administered questionnaires were analyzed according to the intention to treat principle. For each measure, a linear mixed-effects model was used to model the interaction between the two groups. Participant age and gender were set as fixed effects; within-participant variation among time points was set as random effects. The effect size (Cohen’s d) was calculated based on the pooled standard deviations of the two groups at baseline and between each time point.

For social participation, other behavioral changes, and adverse events among hikikomori sufferers, odds ratios were calculated in each time point based on parental reports.

All the analyses were performed using R version 4.1.3 (22), with a significance level of *p* < 0.05.

## Results

### Participant demographics

**FIGURE 1:**
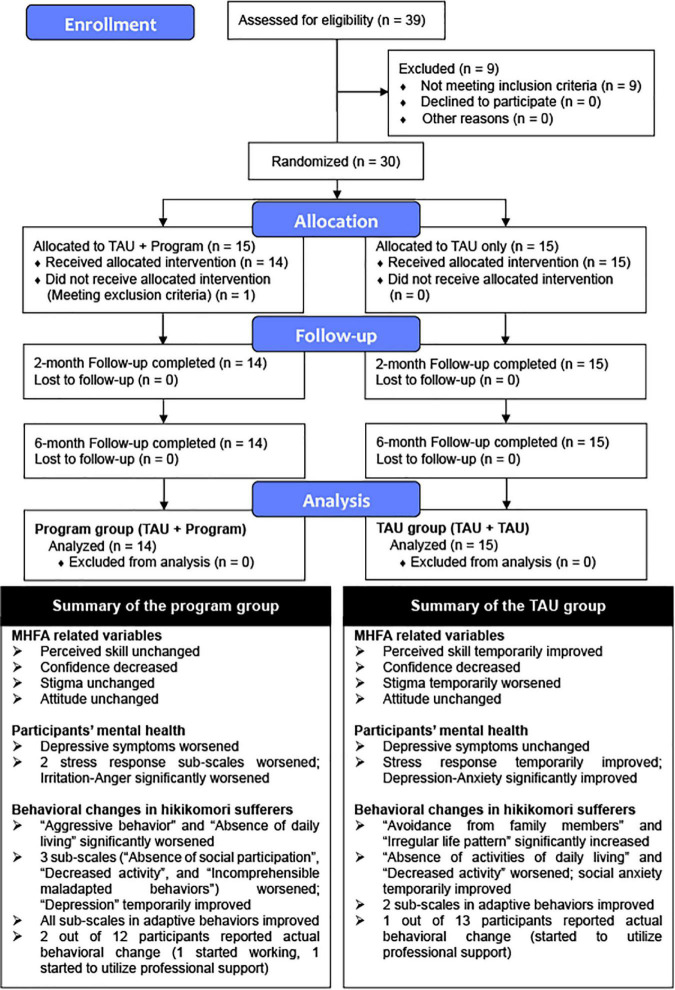
CONSORT flow diagram and summary of the study.

Fourteen participants were assigned to the Program group (Mean age, 67.93; *SD*, 8.56) and 15 to the TAU group (Mean age, 65.60; *SD*, 7.82). Figure 1 shows the CONSORT flow diagram at each stage and summary of the results of the present study. The recruitment rate was 36.25% of our target sample size of 80. This study should be regarded as a pilot RCT for not meeting the requirement based on the sample size estimation. Participants’ and hikikomori sufferers’ demographics are shown in Table 2. There were no significant differences between the Program group and the TAU group in terms of gender and age of participants, or gender, age, and hikikomori duration in hikikomori sufferers.

**TABLE 2:** Baseline demographics of participants and hikikomori sufferers.

	Program (*n* = 14, 12 families)	TAU (*n* = 15, 13 families)	*p*
**Parent (participant)**			
Male %	35.71%	33.33%	0.89^a^
Couple participation %	16.67%	15.38%	1.00^b^
Mean age (SD)	67.93 (8.56)	65.60 (7.82)	0.47^c^
**Hikikomori sufferer**			
Male %	58.33%	61.54%	0.87^a^
Mean age (SD)	36.75 (9.44)	34.08 (8.41)	0.48^c^
Hikikomori duration (SD)	116.08 months (93.40)	163.23 months (101.09)	0.26^c^

^a^Chi-squared test.

^b^Fisher’s exact test.

^c^Welch’s *t*-test.

### Primary

Changes in self-administered questionnaires are shown in Table 3. For the primary outcome, there was no significant change in perceived skill for the Program group; for the TAU group, there was a non-significant temporary increase with a small effect size at T2.

**TABLE 3:** Changes in self-administered questionnaires and behavioral changes in hikikomori sufferers reported by participants.

	T1 (Baseline)	T2	T3	T4	T1 (Baseline)	T2	T3	T4
Variable	Program				TAU			
**MHFA-related variables**								
	*n*, *M* (*SD*)	*n*, *M* (*SD*)	*n*, *M* (*SD*)	*n*, *M* (*SD*)	*n*, *M* (*SD*)	*n*, *M* (*SD*)	*n*, *M* (*SD*)	*n*, *M* (*SD*)
Perceived Skill (Primary outcome)	14, 28.14 (5.71)	14, 28.50 (6.44)	13, 27.23 (6.59)	14 28.29 (5.75)	15, 22.73 (8.03)	14, 22.64 (8.24)	14 26.00 (6.11)	15 23.47 (7.94)
	Effect size d [95% CI]	0.06 [–0.22, 0.33]	–0.20 [–0.57, 0.17]	0.09 [–0.30, 0.48]		0.03 [–0.33, 0.40]	0.27 (small) [–0.37, 0.91]	0.02 [–0.29, 0.34]
Confidence	14, 11.79 (4.57)	14, 10.29 (3.10)	14, 9.50 (3.20)	14, 8.93 (4.22)	15, 10.33 (5.02)	14, 9.86 (5.34)	15, 9.27 (4.88)	15 9.00 (5.24)
	Effect size d [95% CI]	–0.33 (small) [–0.71, 0.04]	–0.54 (medium) [–1.08, 0.01]	–0.63 (medium) [–1.21, –0.04]		–0.01 [–0.30, 0.27]	–0.21 (small) [–0.82, 0.40]	–0.25 (small) [–0.86, 0.36]
Stigma	14, 30.86 (4.17)	14, 31.14 (3.23)	14, 30.57 (3.56)	14, 31.29 (3.75)	15, 29.13 (4.99)	15, 30.00 (5.73)	14, 29.86 (4.90)	15, 29.87 (4.47)
	Effect size d [95% CI]	0.07 [–0.30, 0.44]	–0.07 [–0.60, 0.46]	0.10 [–0.50, 0.71]		0.15 [–0.10, 0.40]	0.26 (small) [–0.02, 0.54]	0.15 [–0.21, 0.51]
Attitude	14, 41.07 (14.71)	13, 40.92 (16.63)	13, 42.46 (17.80)	14, 44.86 (24.36)	15, 33.13 (20.24)	15, 35.87 (17.24)	13, 33.15 (21.65)	15, 30.47 (18.99)
	Effect size d [95% CI]	–0.04 [–0.48, 0.41]	0.07 [–0.27, 0.40]	0.15 [–0.21, 0.52]		0.13 [–0.05, 0.31]	0.02 [–0.36, 0.39]	–0.13 [–0.49, 0.23]
**Participants’ mental health**								
Depressive symptoms (PHQ-9)	14, 4.71 (2.99)	14, 5.50 (3.76)	14, 5.36 (4.70)	14, 5.86 (4.00)	15, 4.20 (5.01)	15, 3.87 (5.80)	15, 4.00 (5.09)	15, 3.47 (4.36)
	Effect size d [95% CI]	0.21 (small) [–0.09, 0.50]	0.15 [–0.33, 0.62]	0.28 (small) [–0.02, 0.59]		–0.06 [–0.25, 0.13]	–0.04 [–0.26, 0.19]	–0.11 [–0.18, –0.03]
Stress response (SRS-18)								
Depression–Anxiety	14, 6.36 (2.74)	14, 7.07 (4.23)	14, 7.29 (4.96)	14, 7.21 (4.62)	15, 5.53 (4.73)	15, 4.87 (4.75)	15, 4.47 (5.03)	15, 3.67 (4.41)
	Effect size d [95% CI]	0.17 [–0.19, 0.52]	0.19 [–0.24, 0.62]	0.20 [–0.27, 0.67]		–0.14 [–0.38, 0.11]	–0.21 (small) [–0.63, 0.21]	–0.39* (small) [-0.76, –0.03]
Irritation–Anger	14, 2.79 (2.27)	14, 3.64 (3.44)	14, 3.64 (2.99)	14, 5.07 (3.71)	15, 3.60 (4.35)	15, 3.67 (3.53)	15, 3.27 (5.38)	15, 2.20 (3.67)
	Effect size d [95% CI]	0.26 (small) [–0.16, 0.67]	0.28 (small) [–0.02, 0.59]	0.66** (medium) [0.13, 1.19]		0.02 [–0.38, 0.41]	–0.06 [–0.51, 0.38]	–0.33 (small) [–0.69, 0.04]
Helplessness	14, 4.79 (2.62)	14, 4.43 (3.20)	14, 6.14 (4.36)	14, 5.50 (2.90)	15, 4.33 (3.40)	15, 3.87 (3.86)	15, 4.27 (4.63)	15, 3.13 (4.05)
	Effect size d [95% CI]	–0.12 [–0.58, 0.35]	0.33 (small) [–0.16, 0.82]	0.25 (small) [–0.20, 0.70]		–0.12 [–0.56, 0.31]	–0.01 [–0.32, 0.29]	–0.30 (small) [–0.64, 0.04]
**Problematic behavior among hikikomori sufferers (HBCL)**								
Aggressive behavior	14, 7.64 (4.43)	14, 8.79 (4.36)	13, 7.54 (4.36)	14, 9.21 (5.82)	15, 4.87 (4.24)	15, 5.73 (4.65)	14, 5.32 (3.82)	15, 4.07 (3.89)
	Effect size d [95% CI]	0.25 (small) [–0.01, 0.51]	0.15 [–0.07, 0.36]	0.23* (small) [0.04, 0.42]		0.19 [–0.14, 0.51]	0.09 [–0.30, 0.48]	–0.19 [–0.62, 0.24]
Social anxiety	14, 7.29 (3.08)	14, 7.36 (2.64)	13, 6.85 (2.28)	14, 7.21 (2.57)	15, 6.87 (2.22)	15, 7.27 (2.67)	14, 7.00 (2.04)	15, 6.23 (2.90)
	Effect size d [95% CI]	0.02 [–0.29, 0.33]	–0.03 [–0.39, 0.33]	–0.02 [–0.23, 0.18]		0.15 [–0.18, 0.48]	0.09 [–0.21, 0.40]	–0.23 (small) [–0.66, 0.20]
Obsessive–compulsive behavior	14, 3.71 (2.89)	14, 4.21 (2.43)	13, 4.08 (2.84)	14, 4.29 (2.71)	15, 3.13 (2.68)	15, 3.00 (2.90)	14, 3.36 (2.97)	15, 3.17 (2.86)
	Effect size d [95% CI]	0.17 [–0.07, 0.41]	0.13 [–0.18, 0.43]	0.20 [–0.13, 0.52]		–0.05 [–0.29, 0.20]	0.09 [–0.14, 0.33]	0.01 [–0.35, 0.37]
Avoidance from family members	14, 6.36 (5.20)	14, 6.86 (5.25)	13, 5.92 (4.25)	14, 7.14 (5.26)	15, 8.87 (2.99)	15, 8.40 (2.63)	14, 8.29 (2.40)	15 7.70 (3.14)
	Effect size d [95% CI]	0.09 [–0.05, 0.23]	0.05 [–0.16, 0.25]	0.14 [–0.00, 0.29]		–0.16 [–0.45, 0.14]	–0.20 [–0.43, 0.04]	–0.37** (small) [-0.69, –0.04]
Depression	14, 3.50 (2.50)	14, 3.71 (2.58)	13, 2.54 (2.37)	14, 3.14 (2.56)	15, 2.53 (3.07)	15, 2.47 (2.45)	14, 2.64 (2.41)	15, 1.93 (2.11)
	Effect size d [95% CI]	0.08 [–0.20, 0.37]	–0.30 (small) [–0.68, 0.08]	–0.14 [–0.49, 0.22]		–0.02 [–0.27, 0.23]	0.04 [–0.18, 0.26]	–0.18 [–0.46, 0.09]
Absence of activities of daily living	14, 6.57 (2.85)	14, 7.64 (4.05)	13, 7.38 (3.77)	14, 8.14 (3.48)	15, 5.73 (3.45)	15, 5.50 (3.44)	14, 6.86 (3.54)	15, 5.80 (3.71)
	Effect size d [95% CI]	0.27 (small) [–0.09, 0.62]	0.22 (small) [–0.09, 0.53]	0.45* (small) [0.16, 0.73]		–0.07 [–0.28, 0.15]	0.25 (small) [–0.00, 0.51]	0.02 [–0.33, 0.36]
Incomprehensible maladapted behavior	14, 2.00 (1.36)	14, 2.93 (1.44)	13, 2.77 (1.67)	14, 2.71 (1.44)	15, 2.07 (1.77)	15, 2.00 (1.71)	14, 2.43 (1.88)	15, 1.83 (1.61)
	Effect size d [95% CI]	0.64 (medium) [–0.06, 1.34]	0.40 (small) [–0.41, 1.20]	0.49 (small) [–0.24, 1.22]		–0.04 [–0.30, 0.22]	0.15 [–0.09, 0.39]	–0.13 [–0.51, 0.24]
Absence of social participation	14, 8.00 (1.13)	14, 7.86 (0.99)	13, 7.62 (1.27)	14, 7.86 (1.36)	15, 7.87 (1.59)	15, 8.10 (1.39)	14, 7.57 (1.64)	15, 8.03 (1.37)
	Effect size d [95% CI]	–0.13 [–0.64, 0.38]	–0.30 (small) [–0.59, –0.00]	–0.11 [–0.77, 0.55]		0.13 [0.01, 0.26]	–0.13 [–0.42, 0.16]	0.09 [–0.01, 0.19]
Decreased activity	14, 5.79 (1.57)	14, 6.21 (1.26)	13, 6.00 (1.88)	14, 6.36 (2.09)	15, 5.73 (1.73)	15, 6.33 (1.92)	14, 6.36 (1.63)	15, 6.10 (1.59)
	Effect size d [95% CI]	0.28 (small) [–0.19, 0.76]	0.22 (small) [–0.44, 0.87]	0.29 (small) [–0.31, 0.90]		0.31 (small) [–0.00, 0.63]	0.32 (small) [–0.16, 0.80]	0.21 (small) [–0.33, 0.76]
Irregular life pattern	14, 4.43 (2.23)	14, 4.64 (2.19)	13, 4.62 (2.56)	14, 4.00 (2.20)	15, 5.87 (1.96)	15, 4.57 (1.78)	14, 5.54 (1.32)	15, 4.90 (1.42)
	Effect size d [95% CI]	0.09 [–0.30, 0.48]	0.03 [–0.18, 0.24]	–0.19 [–0.60, 0.22]		–0.66** (medium) [-1.03, –0.30]	–0.14 [–0.41, 0.13]	–0.53** (medium) [-1.10, 0.03]
**Adaptive behavior among hikikomori sufferers (ABS-H)**								
Interaction	14, 7.71 (4.06)	11, 7.91 (3.85)	14, 9.64 (5.76)	14, 8.79 (5.93)	15, 7.60 (5.56)	12, 6.13 (5.32)	15, 8.13 (5.06)	13, 6.96 (5.83)
	Effect size d [95% CI]	0.25 (small) [–0.36, 0.86]	0.34 (small) [–0.06, 0.74]	0.19 [–0.30, 0.69]		0.04 [–0.43, 0.51]	0.10 [–0.30, 0.50]	0.17 [–0.27, 0.60]
Family	14, 5.00 (3.09)	11, 4.55 (3.03)	14, 4.93 (3.17)	14, 5.93 (3.67)	15, 4.80 (2.79)	12, 4.63 (2.46)	15, 4.70 (2.57)	13, 4.62 (2.79)
	Effect size d [95% CI]	–0.13 [–0.51, 0.24]	–0.02 [–0.31, 0.27]	0.26 (small) [–0.22, 0.74]		0.08 [–0.28, 0.43]	–0.04 [–0.34, 0.27]	0.11 [–0.23, 0.44]
Value	14, 4.07 (2.25)	11, 4.09 (1.88)	14, 3.43 (2.32)	14, 4.86 (3.07)	15, 3.47 (2.99)	12, 3.42 (2.33)	15, 3.90 (2.16)	13, 3.69 (2.20)
	Effect size d [95% CI]	–0.04 [–0.63, 0.55]	–0.27 (small) [–0.73, 0.19]	0.27 (small) [–0.20, 0.74]		0.21 (small) [–0.42, 0.85]	0.16 [–0.36, 0.67]	0.19 [–0.29, 0.67]
Social participation	14, 1.43 (1.72)	11, 1.82 (2.04)	14, 1.57 (1.76)	14, 2.29 (2.99)	15, 1.67 (2.21)	12, 1.08 (1.85)	15, 1.43 (1.78)	13, 2.15 (2.63)
	Effect size d [95% CI]	0.13 [–0.60, 0.86]	0.08 [–0.52, 0.67]	0.32 (small) [–0.26, 0.90]		–0.29 (small) [–0.74, 0.17]	–0.10 [–0.35, 0.14]	0.21 (small) [–0.17, 0.58]
**Behavioral changes in hikikomori sufferers reported by participants**								
	**T1 (Baseline)**		**T2**		**T3**		**T4**	
	*n*/*N*, (%) Program TAU	Odds ratio [95% CI]	*n*/*N*, (%) Program TAU	Odds ratio [95% CI]	*n*/*N*, (%) Program TAU	Odds ratio [95% CI]	*n*/*N*, (%) Program TAU	Odds ratio [95% CI]
Going to work/utilization of support	0/12 (0.00) 0/13 (0.00)	0.93 [0.02, 50.29]	1/12 (8.33) 0/13 (0.00)	0.28 [0.01, 7.67]	1/12 (8.33) 0/13 (0.00)	0.28 [0.01, 7.67]	2/12 (16.67) 1/13 (7.69)	0.42 [0.03, 5.30]
Going outside	7/12 (58.33) 7/13 (53.85)	0.83 [0.17, 4.06]	8/12 (66.67) 7/13 (53.85)	0.58 [0.12, 2.95]	8/12 (66.67) 6/13 (46.15)	0.43 [0.08, 2.17]	8/12 (66.67) 7/13 (53.85)	0.58 [0.12, 2.95]
Avoiding specific area at home	2/12 (16.67) 2/13 (15.38)	0.91 [0.11, 7.72]	1/12 (8.33) 3/13 (23.08)	3.30 [0.29, 37.10]	2/12 (16.67) 4/13 (30.77)	2.22 [0.33, 15.18]	1/12 (8.33) 2/13 (15.38)	2.00 [0.16, 25.41]
Avoiding conversation with family	4/12 (33.33) 4/13 (30.77)	0.89 [0.17, 4.78]	4/12 (33.33) 3/13 (23.08)	0.42 [0.07, 2.36]	3/12 (25.00) 4/13 (30.77)	0.89 [0.17, 4.78]	7/12 (58.33) 2/13 (15.38)	0.13* [0.02, 0.86]
Feeling depressed	8/12 (66.67) 5/13 (38.46)	0.31 [0.06, 1.61]	8/12 (66.67) 6/13 (46.15)	0.43 [0.08, 2.17]	7/12 (58.33) 7/13 (53.85)	0.83 [0.17, 4.06]	8/12 (66.67) 5/13 (38.46)	0.31 [0.06, 1.61]
Feeling irritated	8/12 (66.67) 7/13 (53.85)	0.58 [0.12, 2.95]	8/12 (66.67) 5/13 (38.46)	0.31 [0.06, 1.61]	8/12 (66.67) 6/13 (46.15)	0.43 [0.08, 2.17]	8/12 (66.67) 6/13 (46.15)	0.43 [0.08, 2.17]
Using abusive words	3/12 (25.00) 6/13 (46.15)	2.57 [0.47, 14.10]	5/12 (41.67) 3/13 (23.08)	0.42 [0.07, 2.36]	5/12 (41.67) 3/13 (23.08)	0.42 [0.07, 2.36]	5/12 (41.67) 3/13 (23.08)	0.42 [0.07, 2.36]
Breaking something	0/12 (0.00) 0/13 (0.00)	0.93 [0.02, 50.29]	3/12 (25.00) 0/13 (0.00)	0.10 [0.00, 2.18]	2/12 (16.67) 1/13 (7.69)	0.42 [0.03, 5.30]	3/12 (25.00) 0/13 (0.00)	0.10 [0.00, 2.18]
Domestic violence	0/12 (0.00) 0/13 (0.00)	0.93 [0.02, 50.29]	0/12 (0.00) 0/13 (0.00)	0.93 [0.02, 50.29]	0/12 (0.00) 0/13 (0.00)	0.93 [0.02, 50.29]	0/12 (0.00) 0/13 (0.00)	0.93 [0.02, 50.29]
Suicidal ideation	3/12 (25.00) 1/13 (7.69)	0.25 [0.02, 2.82]	2/12 (16.67) 0/13 (0.00)	0.16 [0.01, 3.60]	1/12 (8.33) 0/13 (0.00)	0.28 [0.01, 7.67]	2/12 (16.67) 0/13 (0.00)	0.16 [0.01, 3.60]
Self-injury	0/12 (0.00) 0/13 (0.00)	0.93 [0.02, 50.29]	0/12 (0.00) 0/13 (0.00)	0.93 [0.02, 50.29]	0/12 (0.00) 0/13 (0.00)	0.93 [0.02, 50.29]	0/12 (0.00) 0/13 (0.00)	0.93 [0.02, 50.29]
Participant’s feeling of danger due to hikikomori sufferer’s behavior	5/12 (41.67) 2/13 (15.38)	0.25 [0.04, 1.69]	4/12 (33.33) 2/13 (15.38)	0.36 [0.05, 2.50]	6/12 (50.00) 3/13 (23.08)	0.30 [0.05, 1.67]	4/12 (33.33) 1/13 (7.69)	0.17 [0.02, 1.78]

Significant results are shown in bold. *p < 0.05, **p < 0.01.

The summary of the results is shown in Figure 1.

### Secondary outcomes

#### (1) Other MHFA-related variables

There was no significant change in confidence in approaching hikikomori sufferers based on MHFA in both groups. However, there was a decrease with small to medium effect size in confidence in the Program group and a decrease with small effect size in the TAU group.

The Program group showed no changes in stigma toward mental health problems. On the other hand, there was a non-significant temporary increase with a small effect size in the TAU group at T2.

There was no significant change in negative attitudes toward hikikomori sufferers in either group.

#### (2) Mental health among participants

There was no significant change in depressive symptoms as measured by the PHQ-9 in either group. However, the Program group showed an increase at T2 and T4, although the effect size was small. For the SRS-18, the Program group showed a significant increase in Irritation–Anger scores at T4, with a medium effect size. Furthermore, there was a non-significant increase in the Irritation–Anger score at T2 and T3 with a small effect size. There was also a non-significant small effect size increase in the Helplessness sub-scale at T3 and T4. These results suggest that mental health in the Program group has worsened. In the TAU group, there was a significant decrease in Depression–Anxiety sub-scales at T4, with a small effect size. Other subscales also showed a non-significant temporary decrease with small effect sizes, suggesting improvement in mental health in the TAU group.

#### (3) Behavioral changes in hikikomori sufferers reported by participants

As for the HBCL, the Program group showed a significant increase in Aggressive behavior and Absence of activities of daily living, with small effect sizes, respectively. Incomprehensible maladapted behavior, Absence of social participation, and Decreased activity showed non-significant increases with small to medium effect sizes. In the TAU group, there was a significant decrease in Avoidance from family members at T4 with a small effect size. There was also a non-significant small effect size decrease in Social anxiety. On the other hand, there was a non-significant increase in Absence of activities of daily living and Decreased activity with a small effect size.

Regarding adaptive behaviors of the hikikomori sufferers as measured by the ABS-H, both groups did not show significant changes. On the other hand, in the Program group, there were increases with small effect size in Interaction at T2 and T3, and in Family and Social participation at T4. Value showed an increase after a decrease with small effect size, suggesting increases in scores for all sub-scales of the ABS-H in Program group. In the TAU group, there was an increase with small effect size in Value at T2, and there was a decrease and an increase with small effect size in Social participation.

Table 3 shows the actual behavioral changes reported by the participants. One of the 12 hikikomori sufferers in the Program group reported the use of a support institution at T2 (8.33%); furthermore, another hikikomori sufferer started to work at T4 (16.67%). For the TAU group, one hikikomori sufferer started the use of a support institution at T4 (7.69%). The rate of going out remained the same for both groups, but the rate of avoidance of specific places in the home temporarily increased in the TAU group. On the other hand, the rate of hikikomori sufferers who were avoiding conversations with family members increased in the Program group at T4, with significant differences between Program group and TAU group.

The baseline rates of depressed feelings, irritability, and suicidal ideation seemed to be higher in the Program group, and the rates in both groups remained almost the same. Domestic violence and self-injury were not reported by either group; the rate of verbal abuse and breaking of property increased in the Program group after the program (T2–T4).

## Discussion

This is the first pilot RCT study to examine the effectiveness of family intervention program for hikikomori. The program seems to have some merits for family members of hikikomori on families’ skill improvement and behavioral changes among the hikikomori sufferers. However, this study was aborted due to the COVID-19, thus we could not draw general conclusions on the effectiveness of the program. Nevertheless, we believe that some important findings were observed in this study. Interestingly, the present study suggested that confidence decreased in both groups. This implies that standard family support (TAU) as well as the present program would decrease confidence of family members approaching hikikomori sufferers. Decrease in confidence in the Program group would be attributed to the increase in revised program content as a result of the addition of active listening and small-step approaches. This might have caused the participants to learn more skills and to feel difficulty, resulting in decreased confidence. In addition, although no serious violence or self-harm was reported in this study, hikikomori sufferers would have shown mental health issues such as depressed mood. It is possible that hasty approaches by family to such hikikomori sufferers may have led to a decreased confidence for not succeeding in affecting behavioral changes of hikikomori sufferers. Increase in avoidance of conversation was suggested among Program group at T4 (6 months later), which may be the result of undesirable family approaches. Monthly follow-up session was conducted in the previous studies; follow-ups after program would be important to evaluate the family’s engagement and to establish skill acquisition. Decreased confidence in the TAU group might be related to different factors as to the Program group. The TAU group showed improved mental health, possibly suggesting that families may be remaining psychologically stable while avoiding confronting hikikomori sufferers. In other words, the family members turn a blind eye and the behavioral changes in hikikomori sufferers would not occur, resulting in the decreased confidence among families. As families’ confidence declines, they might get more reluctant to be in touch with the hikikomori sufferers, leading to prolonged hikikomori. In addition, changes in both problematic and adaptive behaviors were observed in the Program group, suggesting that behavior changes may be more likely to occur by this program. Based on previous studies, we believe that encouraging family to get involved in their hikikomori sufferers is meaningful (6, 7). In addition, some case reports pointed out that interventions encouraging families who are avoiding and keeping distant from hikikomori sufferers to face them may lead to therapeutic changes in family relationships (23, 24). Enabling families to confront their hikikomori sufferers in a safer manner seems to be pivotal.

Therefore, we should revise this intervention program by improving family members’ confidence in engaging with hikikomori sufferers, with safer approaching by families to their hikikomori sufferers. Hikikomori sufferers and their families show diversity; considering their individuality may lead to a safer approach (1). Assessing each parent-child relationship and hikikomori sufferers’ conditions and their surroundings, and gradually increasing interventions according to the situation would be critical. When the family relationship or hikikomori sufferer’s mental health is deteriorating, hasty approaches by family should be retained. To acquire knowledge to assess the situation or condition of hikikomori sufferers and to make approaches according to proper timing, psychoeducation should be contained in the revised program. In this study, aggressive behavior and decreased activity levels were observed in the Program group; introducing these knowledges may be helpful to improve them as well. In addition, we have implemented the role-playing in the present program. A possible revision of the program would be to introduce workshops showing several difficult situations in parent-child interactions and requiring participant to discuss how to respond to such situations. Having a practical outlook on how to respond to troublesome situation would help improve confidence. These revisions would increase the amount of program content. Therefore, the program should be held more frequent, and the amount of content presented in a single session should be reduced and simplified to be more accessible. Based on the parental report in this study, many hikikomori sufferers likely show issues with family relationships and the mental health problems, and prudent response would be required in the family support including group family classes in the present study. Therefore, it will be necessary for program providers to acquire skills such as knowledge of assessment and adequate follow-ups.

Several limitations can be assumed for this study. As noted above, this study recruited only 36.25% of the target sample size, which would have limited its statistical power. Therefore, we have discussed with cautious and proposed future directions. In the future, the study design must be revised to allow for adequate recruitment of participants using online systems even in COVID-19 pandemic (25). In addition, the questionnaire on MHFA-related perceived skills administered for the primary outcome was not confirmed for reliability and validity. Moreover, we could not evaluate skills related to CRAFT for validated questionnaire on CRAFT is not developed as well. Development of a validated skills measurement is warranted and the identification of family factors that can predict behavioral changes in hikikomori sufferers will lead to the establishment of appropriate family support methods. Furthermore, this study was based on family reports of behavioral changes among hikikomori sufferers. Therefore, the report accuracy was considered to be participant-dependent. Accurate observation may be difficult when their hikikomori sufferers are avoiding family, and evaluation from multiple sources, such as interviews with the hikikomori sufferers at the same time will be necessary. In the present study, cases with severe aggression, suicidal thoughts, or severe psychiatric symptoms in both parents and hikikomori sufferers were excluded. Although the mental health of the two groups would be different at baseline, neither group would have included severe mental health problems. Therefore, whether the present program can be applied to more serious cases is unclear. However, interventions like 3-day program, which encourages family members to approach hikikomori sufferers, may need to be implemented more carefully and safely in serious cases. Another limitation is that the program provider and evaluator were the same. The self-administered questionnaire and participants report of specific behavioral changes may have reduced evaluator bias to some extent. In a study design where allocation results are open to both participants and program providers, setting an evaluator independent of the program would be required.

## Data availability statement

The original contributions presented in this study are included in the article/supplementary material, further inquiries can be directed to the corresponding author.

## Ethics statement

The studies involving human participants were reviewed and approved by the Ethics Committee of Kyushu University Hospital. The patients/participants provided their written informed consent to participate in this study.

## Author contributions

HK, HU, SK, TK, TN, and TAK conceived and designed the study. JK performed sample size estimation and conducted assignment of participants. MS, SN, KS, MT, KK, DF, NH, YS, KO, and TK contributed to modifying the program and provided program materials. HK, HU, YH, and TAK recruited the participants and collected data. HK, HU, and TAK implemented the program and analyzed the collected data. HK and TAK wrote the first draft of the manuscript. DF, HU, JK, KS, KK, KO, MT, MS, NH, SK, SN, TN, TK, and YH commented on the manuscript. All authors carefully read the manuscript and approved the final version for submission.

## Footnotes

1
https://center6.umin.ac.jp/cgi-open-bin/ctr/ctr_view.cgi?recptno=R000042510
